# ATX-101, a cell-penetrating protein targeting PCNA, can be safely administered as intravenous infusion in patients and shows clinical activity in a Phase 1 study

**DOI:** 10.1038/s41388-022-02582-6

**Published:** 2022-12-23

**Authors:** Charlotte Rose Lemech, Ganessan Kichenadasse, Jens-Peter Marschner, Konstantinos Alevizopoulos, Marit Otterlei, Michael Millward

**Affiliations:** 1 Scientia Clinical Research, Randwick, Australia and Prince of Wales Clinical School, UNSW Sydney, Sydney, NSW Australia; 2 Southern Oncology Clinical Research Unit, Bedford Park, SA Australia; 3 Therapim Pty Ltd, Southport, QLD Australia; 4 Department of Clinical and Molecular Medicine, Faculty of Medicine and Health Sciences, NTNU Norwegian University of Science and Technology, NO-7491 Trondheim, Norway; 5 Linear Clinical Research & School of Medicine, University of Western Australia, Nedlands, WA Australia

**Keywords:** Drug development, Targeted therapies

## Abstract

Proliferating Cell Nuclear Antigen (PCNA) is a highly conserved protein essential for DNA replication, repair and scaffold functions in the cytosol. Specific inhibition of PCNA in cancer cells is an attractive anti-cancer strategy. ATX-101 is a first-in-class drug targeting PCNA, primarily in cellular stress regulation. Multiple in vivo and in vitro investigations demonstrated anti-cancer activity of ATX-101 in many tumor types and a potentiating effect on the activity of anti-cancer therapies. Healthy cells were less affected. Based on preclinical data, a clinical phase 1 study was initiated. Twenty-five patients with progressive, late-stage solid tumors were treated with weekly ATX-101 infusions at four dose levels (20, 30, 45, 60 mg/m^2^). ATX-101 showed a favorable safety profile supporting that vital cellular functions are not compromised in healthy cells. Mild and moderate infusion-related reactions were observed in 64% of patients. ATX-101 was quickly cleared from blood with elimination half-lives of less than 30 min at all dose levels, probably due to both, a quick cell penetration and peptide digestion in serum, as demonstrated in vivo. No tumor responses were observed but stable disease was seen in 70% of the efficacy population (*n* = 20). Further studies have been initiated to provide evidence of efficacy. Trial registration numbers: ANZCTR 375262 and ANZCTR 375319.

## Introduction

PCNA plays an essential role in orchestrating normal DNA replication, but also acts as a platform for recruiting components of the DNA repair and damage bypass and/or tolerance during replicative stress [1]. PCNA has been shown to have additional scaffold functions in the cytosol, important for regulation of cellular signaling, apoptosis, metabolism, and antitumor immunity [2–7].

The multi-functionality of PCNA-governed regulation under normal and stressed conditions is based on its ability to bind proteins involved in multiple cellular processes. These interactions are mainly mediated via two conserved PCNA-interacting sequences (motifs) found in more than 500 proteins: the PIP-box (PCNA-interacting peptide-box) [8] and APIM (AlkB homolog 2 protein PCNA interacting motif) [9]. These two motifs bind to the same region of PCNA [10]. Affinity differences of these motifs, in combination with dynamically regulated posttranslational modifications (PTM) on PCNA during stress, regulate which proteins bind to PCNA. For example, proteins that bind to PCNA via the high affinity PIP-boxes often control common key processes such as replication, whereas proteins that bind to PCNA via low affinity APIM sequences, require PTM on PCNA and are more important in cellular stress responses, such as DNA repair or regulation of the PI3K/Akt pathway and glycolysis [5, 9, 11–13]. Effective cellular stress responses allow cancer cells to escape anti-cancer therapies; thus, disabling the stress-driven scaffold functions of PCNA is an attractive approach for anti-cancer treatment.

ATX-101 is a novel cell-penetrating APIM-containing peptide, shown to target PCNA and block PCNA-protein interactions [10]. ATX-101 has anti-cancer activity as a single agent in multiple cancer cell lines and cancer models and, in addition, potentiates the activity of multiple other anti-cancer treatments [10, 14–18]. Furthermore, cancer cells that are resistant to chemotherapeutic agents like cisplatin, can be re-sensitized when treated with ATX-101 [15]. The anti-cancer properties of ATX-101 are likely mediated by ATX-101´s ability to alter major cellular signaling pathways [5, 6, 18], reduce central metabolism [19], induce rapid apoptosis [10, 18] and inhibit DNA repair and DNA damage tolerance pathways [9, 13, 20, 21]. PCNA´s role as a scaffold protein in primary metabolism and its impact on glycolytic enzymes and AKT signaling is published by Røst et al. back-to-back to this communication.

To our knowledge, ATX-101 is the only compound that selectively targets PCNA regulatory roles during cellular stress, and a first-in-class compound in clinical development.

Here we report results of the ATX-101 first-in-human study in patients with advanced solid tumors. This open-label, single arm Phase 1 study, consisting of two sub-studies, investigated 4 doses of ATX-101 (20, 30, 45, and 60 mg/m^2^) administered intravenously every week. In a first, dose-escalation study pre-defined dose-limiting toxicities and PK were assessed (for DLT definition see Supplementary Table 1). If, after 6 weeks, no tumor progression was measured, treatment could be continued in a second, long-term follow-up study until disease progression or unacceptable toxicity with the primary objective safety/tolerability. Efficacy was a secondary endpoint in both sub-studies. The studies were registered in the Australian New Zealand Clinical Trials Registry (ANZCTR) under the following IDs: 375262 and 375319. Details on methods are given in the Supplementary information.

## Results and discussion

25 patients were treated. All patients suffered from solid tumors with colorectal (*n* = 4) and non-small cell lung cancer (*n* = 4) being the most frequent diagnoses. Patients were heavily pretreated with half of the patients having received ≥ 4 prior systemic treatment lines. 80% of patients were refractory to the last systemic treatment. Details on patient enrollment, disposition, and demographics are shown in supplementary Fig. 1 and Supplementary Table 2.

### ATX-101 has a favorable safety profile

DLT were not observed, and the maximum tolerated dose was not reached. With exception of one grade 3 adverse event (elevated cholesterol in a patient with hepatocellular carcinoma) only mild and moderate treatment-related adverse events were observed (Table 1). Vital signs, ECG, and laboratory values didn’t show a trend of changes during the treatment with ATX-101. Details on all treatment-emergent adverse events are shown in Supplementary Table 3.

**Table 1:** Incidence of treatment-related TEAE that occurred in more than 1 patient.

Preferred Term, *n* (%) *N*	Cohort 1 20 mg/m^2^	Cohort 2 30 mg/m^2^	Cohort 3 45 mg/m^2^	Cohort 4 60 mg/m^2^	Overall
	(*n* = 8)	(*n* = 3)	(*n* = 4)	(*n* = 10)	(*n* = 25)
Patients with at least 1 event	6 (75.0)	3 (100.0)	4 (100.0)	10 (100.0)	23 (92.0)
Infusion-related reactions	4 (50.0)	3 (100.0)	3 (75.0)	6 (60.0)	16 (64.0)
Fatigue	1 (12.5)	2 (66.7)	1 (25.0)	4 (40.0)	8 (32.0)
Diarrhea	–	1 (33.3)	1 (25.0)	2 (20.0)	4 (16.0)
Dysgeusia	1 (12.5)	0	1 (25.0)	1 (10.0)	3 (12.0)
Anemia	–	–	–	2 (20.0)	2 (8.0)
Hyperglycemia	–	1 (33.3)	–	1 (10.0)	2 (8.0)
Flushing	1 (12.5)	0	0	1 (10.0)	2 (8.0)
Erythema	0	0	1 (25.0)	1 (10.0)	2 (8.0)

All listed TEAEs were CTCAE grade 1 or 2.

Infusion-related reactions (IRR) were identified as the only specific adverse event of ATX-101. Two measures were implemented to prevent the occurrence of IRR: (i) a premedication comprising glucocorticoids, H1 and H2 blockers, diphenhydramine and montelukast, (ii) a stepwise increase of the infusion rate. Despite these measures, IRR were observed in 64% of the patients (Table 2), and in 83% of these patients this was seen on the first infusion day. There was no evidence of dose dependency. IRR were allergic reactions primarily characterized by itching, urticaria and rash. They resolved quickly after treatment interruption or slowing the infusion rate with or without appropriate symptomatic treatment. ATX-101 treatment could be safely restarted in all patients. Notably, the intra-individual recurrence rate of IRR was higher in the 60 mg/m^2^ cohort compared to the other cohorts. This resulted in longer infusion times at the highest dose, i.e., in average 246 min [143-346] (infusion times are shown in Supplementary Table 4). Eventually, the Safety Monitoring Committee recommended to stop the dose escalation after completion of the 60 mg/m^2^ cohort despite lack of safety concerns. The reason is that infusion times of more than 4–5 h can hardly be managed in an outpatient clinic. Consequently, the recommended phase 2 dose (RP2D) for ATX-101 monotherapy was defined as 60 mg/m^2^.

**Table 2:** Incidence and CTCAE grading of infusion-related reactions (IRR).

CTCAE grade, *n* (%) *N*	Cohort 1 20 mg/m^2^	Cohort 2 30 mg/m^2^	Cohort 3 45 mg/m^2^	Cohort 4 60 mg/m^2^	Overall
	(*n* = 8)	(*n* = 3)	(*n* = 4)	(*n* = 10)	(*n* = 25)
Patients with at least one event	4 (50.0) 9	3 (100.0) 5	3 (75.0) 11	6 (60.0) 37	16 (64.0) 62
Grade 1 (mild)	2 (25.0) 6	3 (100.0) 4	1 (25.0) 3	3 (30.0) 4	9 (36.0) 17
Grade 2 (moderate)	3 (37.5) 3	1 (33.3) 1	3 (75.0) 8	6 (60.0) 33	13 (52.0) 45
Grade 3 (severe)	–	–	–	–	–
Grade 4 (life threatening)	–	–	–	–	–

n: number of patients with IRR; N: total number of IRRs.

The reason for IRR has not been completely clarified. In vitro data from mast cells and data generated in dogs suggest a correlation of IRR with a transient increase in histamine levels induced by ATX-101 (unpublished data). Such increases of histamine could not be reproduced in this study because data from only 3 patients experiencing an IRR were available. Further investigations are ongoing in current clinical studies. The development of anti-drug antibodies is deemed to be unlikely because of the above-mentioned preclinical data and clinical data showing that IRR occurred in most patients during the first infusion and did not exacerbate during the treatment. In addition, peptide with cell penetrating, cationic parts are known to cause histamine release [22].

### ATX-101 is rapidly cleared from plasma indicating quick cell penetration

**Fig. 1:**
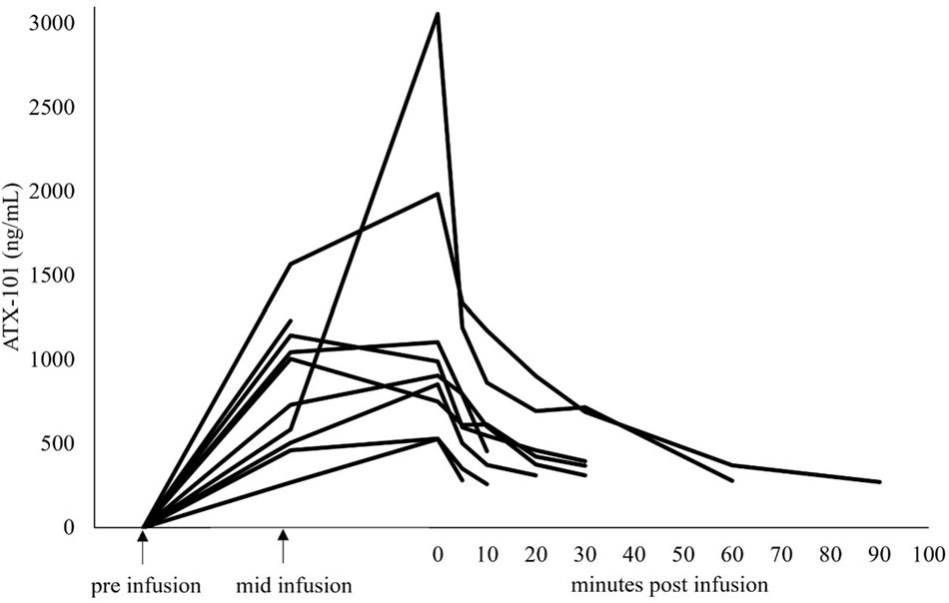
Individual plasma concentration–time curves for patients treated with 60 mg/m^2^ (recommended phase 2 dose). Infusion times were in the range of 115–346 min. For the sake of simplicity, the infusion time has been arbitrarily shortened and presented uniformly for all patients.

ATX-101 was rapidly cleared from blood with a half-life shorter than 30 min in all dosing groups. PK parameters (Table 3) showed that maximum plasma concentrations (*C*_max_) and area under the curve (AUC) were dose dependent. *C*_max_ were reached at mid or directly at the end of infusion. However, *C*_max_ values were very different for the individual patients. For instance, in the highest dosing group *C*_max_ values varied between 527 and 3056 ng/mL (mean 1,150). One contributor to this variation is probably the infusion time in the context of the short half-life of ATX-101 in plasma; as longer the infusion time is, as more ATX-101 is cleared already during the infusion and this results in smaller *C*_max_ values. Therefore, despite a dose dependence, the *C*_max_ appear to be comparable for the 45 and 60 mg/m^2^ cohorts due to the different average infusion times: 142 and 246 min, respectively (Supplementary Table 4). Figure 1 shows the individual plasma concentration-time curves for the highest dosing cohort. The quick clearance of ATX-101 from plasma is in accordance with preclinical data showing rapid cell penetration and degradation in serum, but still good tissue distribution [10, 15]. In cell cultures, activity, characterized by inhibition of growth and increased apoptosis, was observed for up to five days after addition of the peptide [10]. Of note, no full-length peptide was found in the culture media 1–2 h after the peptide addition (unpublished data). Based on these data, it is concluded that the plasma concentration doesn’t reflect the biological availability of ATX-101 in patients.

**Table 3:** Summary of PK parameters (primary PK population).

	ATX-101 20 mg/m^2^		ATX-101 30 mg/m^2^		ATX-101 45 mg/m^2^		ATX-101 60 mg/m^2^	
Parameter (unit)	*n*	Value	*n*	Value	*n*	Mean (SD)	*n*	Mean (SD)
AUC_0-inf_ (h × ng/mL)	0	N/A	1	2080	4	2200 (591)	6	3700 (1220)
AUC_0-t_ (h × ng/mL)	1	666	1	1900	4	1980 (427)	8	3320 (1260)
*C*_max_ (ng/mL)	1	468	1	720	4	1150 (166)	8	1320 (815)
*k*_el_ (/h)	0	N/A	1	1.81	4	2.25 (1.57)	6	1.51 (0.46)
CL (mL/h/m^2^)	0	N/A	1	14,400	4	21,500 (6120)	6	17,800 (5880)
Vz (mL/m^2^)	0	N/A	1	7970	4	13,100 (6830)	6	12,900 (5520)
MRT_0-inf_ (h)	0	N/A	1	0.611	4	0.889 (0.393)	6	1.54 (0.66)
*C*_max_/*D* (m^2^ × ng/mL/mg)	1	23.4	1	24	4	25.6 (3.68)	8	22 (13.6)
AUC_0-inf_/*D* (h × [ng/mL] × m^2^/mg)	0	N/A	1	69.5	4	49.3 (13.1)	6	61.7 (20.4)
AUC_0-t_/*D* (h × [ng/mL] × m^2^/mg)	1	33.3	1	63.5	4	44 (9.49)	8	55.4 (21.1)
*t*_1/2 el_ (h) Median [Min;Max]	0	N/A	1	0.38 [N/A]	4	0.45 [0.19;0.87]	6	0.48 [0.31;0.70]
*t*_max_ (h) Median [Min;Max]	1	2.02 [N/A]	1	2.98 [N/A]	4	2.37 [1.32;2.82]	8	4.75 [1.60;9.12]
*t*_last_ (h) Median [Min;Max]	1	2.17 [N/A]	1	3.32 [N/A]	4	2.77 [2.25;3.82]	8	5.13 [3.23;9.62]

*AUC* area under the curve (0-t: from infusion start to the last measurable concentration, *0-inf* from infusion start extrapolated to infinity), *C*_*ma*x_ maximum plasma concentration, *k*_el_ elimination rate constant, *CL* clearance, *Vz* volume of distribution, *MRT* mean residence time, *D* dose, *t*_*1/2 el*_ elimination half-life, *t*_*max*_ time from infusion start to maximum plasma concentration, *t*_*last*_ time from infusion start to last plasma drug concentration assessment, *SD* standard deviation.

### ATX-101 showed antitumor activity

Fourteen (70%) of 20 patients belonging to the Efficacy Population had stable disease at the end of the dose escalation study. Twelve patients (60%) continued treatment in the long-term follow-up study. At treatment discontinuation, after a median time of 18 weeks [7–74], 5 of 12 patients (41.7%) had stable disease, and 7 patients (58.3%) had progressive disease (treatment duration is shown in Supplementary Fig. 2). No partial or complete responses were observed. Overall, in 8 patients (40% of Efficacy Population) disease stabilization was observed over a period of at least 4.1 months. The longest disease stabilization was observed in a patient with uterine leiomyosarcoma. She was treated over 17 months and remained stable after 29 months when she was lost to follow-up. Disease stabilization can probably be attributed to ATX-101, considering that 96% of the patients had progressive disease at study entry, 80% were refractory to the most recent systemic treatment, and most patients were heavily pretreated.

Of note, the above-mentioned disease stabilization was independent of the dose and was observed in 50%, 33.3%, 50% and 33.3% of patients belonging to the Efficacy Population in Cohorts 1–4, respectively.

Due to the heterogenous patient population with different tumor entities characterized by differences in prognoses, prior treatment lines and life expectancies, no meaningful efficacy or efficacy-dose relationship evaluations were possible. So far, no clinical pharmacodynamic (PD) markers have been identified that help to describe the mode of action in patients and to support the clinical dose finding. Biopsies collected in an ongoing study will be analyzed in order to identify potential biomarkers. Blood samples have also been collected at all visits for all patients in this Phase 1 study for use in future analysis.

## Conclusion

This study showed that ATX-101 was well tolerated when administered as weekly infusions. IRR were a typical side effect occurring in the majority of patients. The RP2D was set at 60 mg/m^2^. Exploratory efficacy data suggest activity of ATX-101 in terms of disease stabilization in patients with advanced solid tumors. Neither safety nor efficacy appeared to be dose dependent. Based on these results and preclinical data showing that ATX-101 reduce primary metabolism, alter cellular signaling and potentiate multiple anticancer therapies, a Phase 1b/2a proof of concept study investigating ATX-101 in combination with platinum-based chemotherapy was started in patients with platinum sensitive ovarian cancer (NCT04814875).

## Supplementary information

Methods

Figure s1

Figure s2

Table s1

Table s2

Table s3

Table s4
