# Supplementary material for: ATX-101, a cell-penetrating protein targeting PCNA, can be safely administered as intravenous infusion in patients and shows clinical activity in a Phase 1 study
Source: Oncogene. 2022 Dec 23;42(7):541–4. doi: 10.1038/s41388-022-02582-6 (PMC9918429; doi:10.1038/s41388-022-02582-6)
Supplement: Supplementary file 1 — Methods [file 41388_2022_2582_MOESM1_ESM.docx]

**Methods**

**Patients**

The study was conducted at four Australian sites between August 2018 and November 2021. Patients suffering from advanced solid tumors were eligible if they were ≥ 18 years of age and signed the informed consent. Patients had to have an ECOG performance status of 0-2 and a life expectancy of at least 3 months. Patients were excluded in case of cardiac failure, clinically significant cardiac disease, other significant acute or chronic diseases (with exception of the study indication), or CNS metastases.

**Study Design**

This Phase 1, open-label, single arm, first-in-human study consisted of two sub-studies. Study ATX101-01 was a dose escalation study in accordance with the traditional 3+3 escalation design^1^. Patients were treated over 6 weeks when the first tumor assessment was done. In case of disease stabilization or tumor response, patients could continue treatment in the long-term follow-up study ATX101-02. The data analysis has been combined for both studies.

ATX-101 was administered weekly as intravenous infusion.

Due to the occurrence of infusion related reactions (IRR) in the first patient, a premedication was implemented for subsequent patients. Premedication was optimized by the Safety Monitoring Committee (SMC) during the study resulting in the final recommendation to administer corticosteroids, paracetamol, H1 and H2 inhibitors. This medication became standard practice in Cohort 3. Montelukast (anti-leukotriene) was added in Cohort 4. In addition, on each infusion day, the infusion rate had to be increased in 5 steps from 5 mg/h to a maximum of 120 mg/h.

Treatment could be continued until disease progression, unacceptable toxicity, withdrawal of consent or non-compliance.

ATX-101 was administered in 4 dosing cohorts. The starting dose was 20 mg/m², escalated to 30 mg/m², 45 mg/m² and 60 mg/m². An SMC, comprising all principal investigators and medical monitors of sponsor and CRO, recommended dose changes and modifications after the end of each completed cohort and during the entire study.

**Study Assessments**

Toxicities were graded as mild, moderate, severe, life threatening or related to death using the Common Terminology Criteria for Adverse Events (CTCAE) Version 4.03. Safety assessments included the incidence of dose limiting toxicities (DLTs), treatment-emergent adverse events (TEAE), laboratory parameters, ECG, performance status, physical examinations. DLT were defined upfront, the DLT period included the first three treatment weeks.

Blood samples for a complete pharmacokinetic (PK) profile were collected on the first infusion day. On days 8, 15, and 36 samples were collected during the infusion only. Plasma trough levels were assessed on Days 3 and Day 43.

Tumors were assessed by investigators according to RECIST V1.1 at baseline, after 6 weeks and every 3 months thereafter.

**Statistical Analysis**

All data were summarized descriptively at each scheduled time point using descriptive summary statistics. Categorical data were summarized as frequency counts and percentages at each scheduled time point. For all percentage calculations, the denominator was the number of patients in the relevant population. Patient populations defined for statistical analyses are described in supplementary Figure 1.

PK parameters were computed from the individual plasma ATX-101 concentrations by a non-compartmental approach using Phoenix WinNonlin (Version 8.3) software.

**Study Oversight**

All patients provided written informed consent before undergoing any study-specific procedures. The study was conducted in accordance with the principles of the Declaration of Helsinki and local laws and regulations. The principles outlined in the Good Clinical Practice International Conference on Harmonization Tripartite Guideline were considered. The protocol was approved by the national and participating-institution independent ethics committees. The studies were registered in the Australian New Zealand Clinical Trials Registry (ANZCTR) under the following IDs: 375262 and 375319.

1. Le Tourneau C, Lee JJ, Siu LL. Dose escalation methods in phase I cancer clinical trials. *J Natl Cancer Inst*. May 20 2009;101(10):708-20. doi:10.1093/jnci/djp079
