# Supplementary material for: ATX-101, a cell-penetrating protein targeting PCNA, can be safely administered as intravenous infusion in patients and shows clinical activity in a Phase 1 study
Source: Oncogene. 2022 Dec 23;42(7):541–4. doi: 10.1038/s41388-022-02582-6 (PMC9918429; doi:10.1038/s41388-022-02582-6)
Supplement: Supplementary file 2 — Figure s1 [file 41388_2022_2582_MOESM2_ESM.docx]

**Figure s1: Patient enrolment and disposition**

**25 Patients**

**Dose Escalation**

**20 mg/m^2^ ATX-101**

- Enrolled: n=8
- Terminated early: n=4, due to
- PD: n=2
- TEAE: n=1
- Withdrawal of consent: n=1
- Safety Population: n=8
- Evaluable for DLT: n=4
- Efficacy Population: n=6
- Primary PK Population: n=1
- Secondary PK Population: n=7

**Long-term Follow-up**

**20 mg/m^2^ ATX-101**

- Enrolled: n=4
- Safety Population: n=4
- Efficacy Population: n=4

**Dose Escalation**

**30 mg/m^2^ ATX-101**

- Enrolled: n=3
- Terminated early: n=0
- Safety Population: n=3
- Evaluable for DLT: n=3
- Efficacy Population: n=3
- Primary PK Population: n=1
- Secondary PK Population: n=3

**Long-term Follow-up**

**30 mg/m^2^ ATX-101**

- Enrolled: n=1
- Safety Population: n=1
- Efficacy Population: n=1

**Dose Escalation**

**45 mg/m^2^ ATX-101**

- Enrolled: n=4
- Terminated early: n=2, due to
- PD: n=1
- Other: n=1
- Safety Population: n=4
- Evaluable for DLT: n=3
- Efficacy Population: n=2
- Primary PK Population: n=4
- Secondary PK Population: n=4

**Long-term Follow-up**

**45 mg/m^2^ ATX-101**

- Enrolled: n=2
- Safety Population: n=2
- Efficacy Population: n=2

**Dose Escalation**

**60 mg/m^2^ ATX-101**

- Enrolled: n=10
- Terminated early: n=4, due to
- PD: n=1
- TEAE: n=1
- Other: n=2
- Safety Population: n=10
- Evaluable for DLT: n=5
- Efficacy Population: n=9
- Primary PK Population: n=8
- Secondary PK Population: n=10

**Long-term Follow-up**

**60 mg/m^2^ ATX-101**

- Enrolled: n=5
- Safety Population: n=5
- Efficacy Population: n=5

Safety Population: patients who received any amount of study drug

Efficacy Population: patients with at least one tumor assessment (RECIST) after treatment start

Primary PK Population: patients with at least 2 measurable plasma concentration time points after the end of infusion on D1

Secondary PK Population: patients with at least 1 measurable plasma concentration time point during and after infusion

PD: Progressive Disease; TEAE: treatment emergent adverse event
