# Supplementary material for: ATX-101, a cell-penetrating protein targeting PCNA, can be safely administered as intravenous infusion in patients and shows clinical activity in a Phase 1 study
Source: Oncogene. 2022 Dec 23;42(7):541–4. doi: 10.1038/s41388-022-02582-6 (PMC9918429; doi:10.1038/s41388-022-02582-6)
Supplement: Supplementary file 3 — Figure s2 [file 41388_2022_2582_MOESM3_ESM.docx]

**Figure s2: Tumor type and treatment duration sorted by length for the individual patients (Intent to Treat Population) including radiological tumor status at end of treatment**

**Tumor Status at EOI**

SD: Stable Disease

PD: Progressive Disease

N.E. Not Evaluable (RECIST)

**Reason for EOT if not PD**

WoC: Withdrawal of Consent

AE: Adverse Event

CP: Clinical Progression


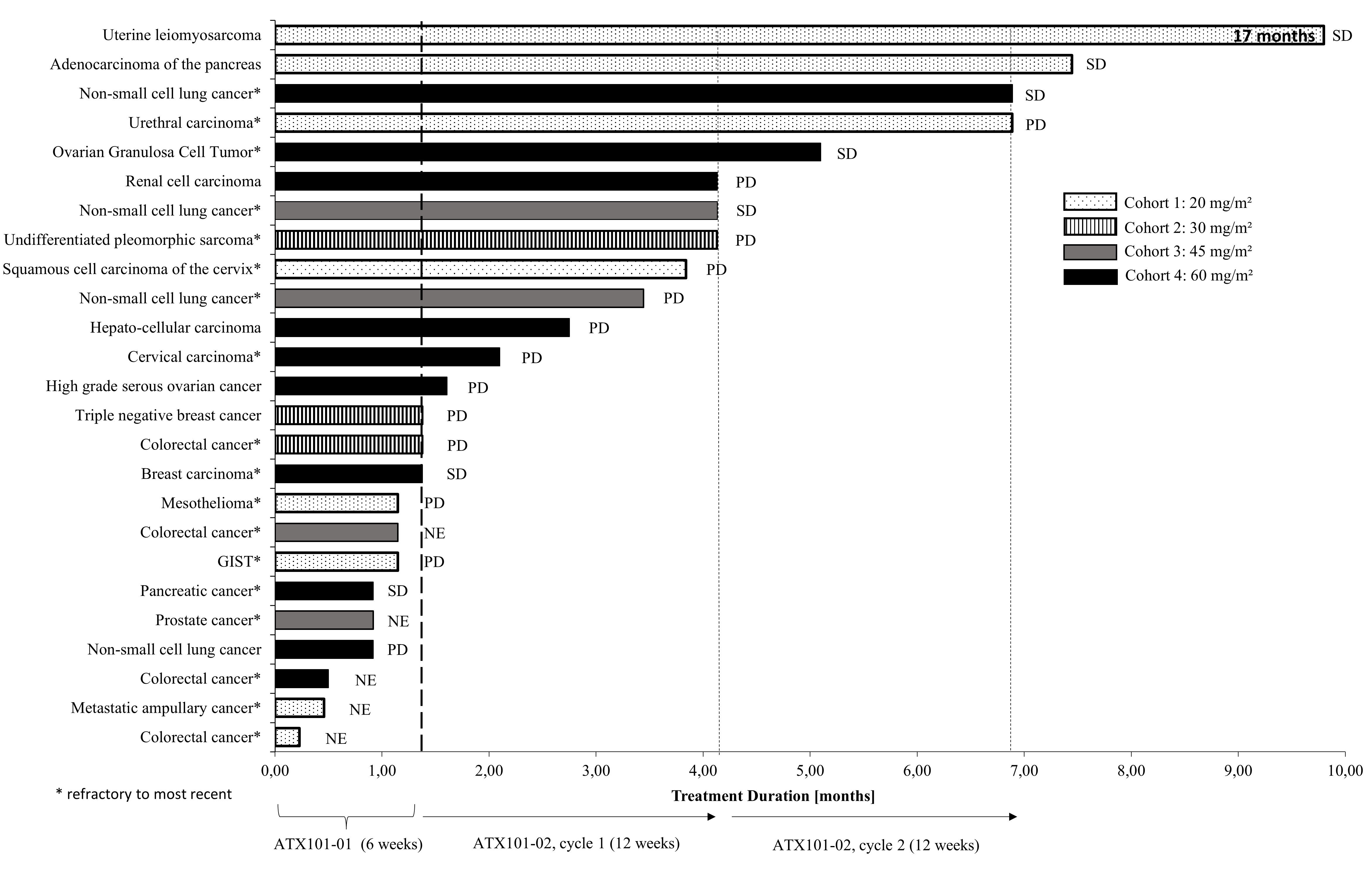


NE: not evaluable; PD: progressive disease; SD: stable disease
