# Supplementary material for: ATX-101, a cell-penetrating protein targeting PCNA, can be safely administered as intravenous infusion in patients and shows clinical activity in a Phase 1 study
Source: Oncogene. 2022 Dec 23;42(7):541–4. doi: 10.1038/s41388-022-02582-6 (PMC9918429; doi:10.1038/s41388-022-02582-6)
Supplement: Supplementary file 4 — Table s1 [file 41388_2022_2582_MOESM4_ESM.docx]

| **Table s1: Dose Limiting Toxicities (DLT)** |  |
| --- | --- |
| A DLT may include any of the following ATX-101 related toxicities:   - All Grade 5 treatment related AEs - ≥Grade 3 non-hematologic treatment related toxicities (except for, nausea, vomiting, anorexia and hypersensitivity reactions), not reversible within 72 hours to Grade 2 or baseline grade with adequate medical therapy - Any Grade 4 hematologic treatment related AEs will be considered DLTs with the exception of:   - Grade 4 neutropenia lasting < 5 days when treated with adequate medical therapy   - Grade 4 neutropenia lasting ≤ 48 hours that is not associated with fever or other clinically significant symptoms   - Platelet counts ≤ 25 000 cells per mm^3^   - Other Grade 4 hematologic AEs that last less than 7 days - Treatment delay greater than 2 weeks due to treatment-related toxicity. | |
