# Supplementary material for: ATX-101, a cell-penetrating protein targeting PCNA, can be safely administered as intravenous infusion in patients and shows clinical activity in a Phase 1 study
Source: Oncogene. 2022 Dec 23;42(7):541–4. doi: 10.1038/s41388-022-02582-6 (PMC9918429; doi:10.1038/s41388-022-02582-6)
Supplement: Supplementary file 5 — Table s2 [file 41388_2022_2582_MOESM5_ESM.docx]

| **Table s2: Demographics and baseline characteristics** | | | | | |  |
| --- | --- | --- | --- | --- | --- | --- |
| **Characteristics** | **Cohort 1**  **20 mg/m^2^** | **Cohort 2**  **30 mg/m^2^** | **Cohort 3**  **45 mg/m^2^** | **Cohort 4**  **60 mg/m^2^** | **Overall (n=25)** |  |
| Number of patients | 8 | 3 | 4 | 10 | 25 |  |
| Median age, years (range) | 60 (48-73) | 50 (37-73) | 64 (31-69) | 59 (48-70) | 59 (31-73) |  |
| Sex, n (%) |  |  |  |  |  |  |
| Male | 6 (75.0%) | 2 (66.7%) | 2 (50.0%) | 3 (30.0%) | 13 (52.0%) |  |
| Female | 2 (25.0%) | 1 (33.3%) | 2 (50.0%) | 7 (70.0%) | 12 (48.0%) |  |
| Race, n (%) |  |  |  |  |  |  |
| Caucasian | 6 (75.0%) | 3 (100.0%) | 3 (75.0%) | 9 (90.0%) | 21 (84.0%) |  |
| Asian | 2 (25.0%) | - | 1 (25.0%) | 1 (10.0%) | 4 (16.0%) |  |
| ECOG Performance Status, n (%) |  |  |  |  |  |  |
| 0 | 6 (75.0%) | 2 (66.7%) | 2 (50.0%) | 4 (40.0%) | 14 (56.0%) |  |
| 1 | 1 (12.5%) | 1 (33.3%) | 1 (25.0%) | 5 (50.0%) | 8 (32.0%) |  |
| 2 | 1 (12.5%) |  | 1 (25.0%) | 1 (10.0%) | 3 (12.0%) |  |
| Cancer type, n (%) | |  |  |  |  |  |
| Colorectal | | 1 | 1 | 1 | 1 | 4 (16.0%) |
| Non-small cell lung | | - | - | 2 | 2 | 4 (16.0%) |
| Pancreatic (incl. ampullary) | | 2 | - | - | 1 | 3 (12.0%) |
| Breast | | - | 1 | - | 1 | 2 (8.0%) |
| Cervical | | 1 | - | - | 1 | 2 (8.0%) |
| Ovarian | | - | - | - | 2 | 2 (8.0%) |
| Sarcoma | | 1 | 1 | - | - | 2 (8.0%) |
| GI stromal | | 1 | - | - | - | 1 (4.0%) |
| Hepato-cellular | | - | - | - | 1 | 1 (4.0%) |
| Mesothelioma | | 1 | - | - | - | 1 (4.0%) |
| Prostate | | - | - | 1 | - | 1 (4.0%) |
| Renal cell | | - | - | - | 1 | 1 (4.0%) |
| Urethral | | 1 | - | - | - | 1 (4.0%) |
| Metastatic disease, n (%) | |  |  |  |  |  |
| M0 | | - | - | - | 1 | 1 (4.0%) |
| M1 | | 8 (100.0%) | 2 (66.7%) | 4 (100.0%) | 9 (90.0%) | 23 (92.0%) |
| No data | | - | 1 (33.3%) | - | - | 1 (4.0%) |
| Median number of prior systemic therapies (range) | 3.5 (1-5) | 3.0 (3-5) | 5.5 (3-6) | 4.0 (2-10) | 4 (1-10) |  |
| Outcome of last therapy, n (%) |  |  |  |  |  |  |
| Stop due to PD | 6 (75.0%) | 2 (66.7%) | 4 (100.0%) | 8 (80.0%) | 20 (80.0%) |  |
| Completed regimen | 2 (25.0%) | - | - | 1 (10.0%) | 3 (12.0%) |  |
| Stop due to adverse event | - | 1 (33.3%) | - | - | 1 (4.0%) |  |
| No data | - | - | - | 1 | 1 (4.0%) |  |
