# Supplementary material for: ATX-101, a cell-penetrating protein targeting PCNA, can be safely administered as intravenous infusion in patients and shows clinical activity in a Phase 1 study
Source: Oncogene. 2022 Dec 23;42(7):541–4. doi: 10.1038/s41388-022-02582-6 (PMC9918429; doi:10.1038/s41388-022-02582-6)
Supplement: Supplementary file 6 — Table s3 [file 41388_2022_2582_MOESM6_ESM.docx]

| **Table s3: Incidence of treatment emergent adverse events that occurred in more than 2 patients** | | | | | | | | | | | | |  |
| --- | --- | --- | --- | --- | --- | --- | --- | --- | --- | --- | --- | --- | --- |
| **Preferred Term, n (%)** | **Cohort 1 (n=8)**  **20 mg/m^2^** | | **Cohort 2 (n=3)**  **30 mg/m^2^** | | **Cohort 3 (n=4)**  **45 mg/m^2^** | | **Cohort 4 (n=10)**  **60 mg/m^2^** | | | **Overall (n=25)** | | | |
|  | Any Grade | Grade 3 | Any Grade | Grade 3 | Any Grade | Grade 3 | Any Grade | Grade 3 | | Any Grade | Grade 3 | | |
| *Patients with at least one event* | *8 (100.0)* | *4 (50.0)* | *3 (100.0)* | *2 (66.7)* | *4 (100.0)* | *1 (25.0)* | *10 (100.0)* | | *8 (80.0)* | *25 (100.0)* | | *15 (60.0)* | |
| Infusion related reactions | 4 (50.0) | - | 3 (100.0) | - | 3 (75.0) | - | 6 (60.0) | | - | 16 (64.0) | | - | |
| Fatigue | 2 (25.0) | - | 2 (66.7) | - | 3 (75.0) | 1 (25.0) | 5 (50.0) | | - | 12 (48.0) | | 1 (4.0) | |
| Diarrhea | - | - | 1 (33.3) | - | 2 (50.0) | - | 4 (40.0) | | - | 7 (28.0) | | - | |
| Anemia | 2 (25.0) | 2 (25.0) | - | - | 1 (25.0) | 1 (25.0) | 5 (50.0) | | 1 (10.0) | 8 (32.0) | | 4 (16.0) | |
| Abdominal pain | 4 (50.0) | - | - | - | - | - | 1 (10.0) | | - | 5 (20.0) | | - | |
| Nausea | 1 (12.5) | - | - | - | 1 (25.0) | - | 2 (20.0) | | - | 4 (16.0) | | - | |
| Pyrexia | 2 (25.0) | - | 1 (33.3) | - | 1 (25.0) | - | - | | - | 4 (16.0) | | - | |
| Abdominal pain upper | 1 (12.5) | 1 (12.5) | 1 (33.3) | - | - | - | 1 (10.0) | | - | 3 (12.0) | | 1 (4.0) | |
| Dysgeusia | 1 (12.5) | - | - | - | 1 (25.0) | - | 1 (10.0) | | - | 3 (12.0) | | - | |
| Erythema | - | - | 1 (33.3) | - | 1 (25.0) | - | 1 (10.0) | | - | 3 (12.0) | | - | |
| Headache | 1 (12.5) | - | 1 (33.3) | - | - | - | 1 (10.0) | | - | 3 (12.0) | | - | |
| Hyperglycemia | - | - | 1 (33.3) | - | - | - | 2 (20.0) | | 2 (20.0) | 3 (12.0) | | 2 (8.0) | |
| Insomnia | 1 (12.5) | - | - | - | 1 (25.0) | - | 1 (10.0) | | - | 3 (12.0) | | - | |
| Upper respiratory tract infection | 1 (12.5) | - | 1 (33.3) | - | - | - | 1 (10.0) | | - | 3 (12.0) | | - | |
| Vascular access site bruising | 1 (12.5) | - | - | - | - | - | 2 (20.0) | | - | 3 (12.0) | | - | |
| CTCAEs grade 4/5 were not observed | | | | | | | | | | | | |  |
