# Supplementary material for: ATX-101, a cell-penetrating protein targeting PCNA, can be safely administered as intravenous infusion in patients and shows clinical activity in a Phase 1 study
Source: Oncogene. 2022 Dec 23;42(7):541–4. doi: 10.1038/s41388-022-02582-6 (PMC9918429; doi:10.1038/s41388-022-02582-6)
Supplement: Supplementary file 7 — Table s4 [file 41388_2022_2582_MOESM7_ESM.docx]

| **Table s4: Infusion times on Day 1** | | | | |  |
| --- | --- | --- | --- | --- | --- |
| **Characteristics** | **Cohort 1**  **20 mg/m^2^** | **Cohort 2**  **30 mg/m^2^** | **Cohort 3**  **45 mg/m^2^** | **Cohort 4**  **60 mg/m^2^** | |
| Number of patients | 8* | 3 | 4 | 10 | |
| Individual infusion times (min) | 130  122  122  121  115  120  122 | 179  295  136 | 163  169  115  120 | 190  160  346  305  190  143  221  331  288  282 | |
| Average infusion time (min) | 122 | 203 | 142 | 246 | |
| * Infusion of one patient was discontinued 5 minutes after treatment start. | | | | | |
